# Type 2 Diabetes Increases Risk of Unfavorable Survival Outcome for Postoperative Ischemic Stroke in Patients Who Underwent Non-cardiac Surgery: A Retrospective Cohort Study

**DOI:** 10.3389/fnagi.2021.810050

**Published:** 2022-01-11

**Authors:** Faqiang Zhang, Yulong Ma, Yao Yu, Miao Sun, Hao Li, Jingsheng Lou, Jiangbei Cao, Yanhong Liu, Mu Niu, Long Wang, Weidong Mi

**Affiliations:** ^1^School of Medicine, Nankai University, Tianjin, China; ^2^Anesthesia and Operation Center, The First Medical Center, Chinese PLA General Hospital, Beijing, China; ^3^Department of Neurology, The Affiliated Hospital of Xuzhou Medical University, Xuzhou Medical University, Xuzhou, China; ^4^Department of Pain Medicine, The First Medical Center, Chinese PLA General Hospital, Beijing, China

**Keywords:** type 2 diabetes mellitus (type 2 DM), overall survival, perioperative stroke, postoperative complications, large hemispheric infarction (LHI)

## Abstract

**Objective:** Diabetes mellitus (DM) has been critically associated with unfavorable outcomes in the general population. We aimed to investigate the association between type 2 DM and long-term survival outcomes for postoperative ischemic stroke in patients who underwent non-cardiac surgery.

**Research Design and Methods:** This was a retrospective cohort study of patients with non-cardiac surgery who had suffered from postoperative ischemic stroke between January 2008 and August 2019. Diabetic individuals were included in postoperative ischemic stroke patients with the DM group. The outcome of interest was long-term overall survival (OS). We conducted propensity score matching (PSM) and inverse probability treatment weighting (IPTW) to adjust for baseline characteristic differences between groups. Multivariate Cox regression analysis with stepwise selection was used to calculate the adjusted hazard ratio (HR) of OS and type 2 DM.

**Results:** During a median follow-up of 46.2 month [interquartile range (IQR), 21.1, 84.2], 200 of 408 patients (49.0%) died. The OS rates at 3, 5, and 10 years were significantly lower for postoperative ischemic stroke patients with DM than those without DM (3 years OS: 52.2 vs. 69.5%, *p* < 0.001; 5 years OS: 41.6 vs. 62.4%, *p* < 0.001; 10 years OS: 37.2 vs. 56.6%, *p* < 0.001). All covariates were between-group balanced after using PSM or IPTW. The postoperative ischemic stroke patients with type 2 DM had a shortened OS in primary analysis (HR: 1.947; 95% CI: 1.397–2.713; *p* < 0.001), PSM analysis (HR: 2.190; 95% CI: 1.354–3.540; *p* = 0.001), and IPTW analysis (HR: 2.551; 95% CI: 1.769–3.679; *p* < 0.001).

**Conclusion:** Type 2 DM was associated with an unfavorable survival outcome for postoperative ischemic stroke in patients who underwent non-cardiac surgery. When postoperative ischemic stroke co-occurred with type 2 DM, the potential synergies would have multiplicative mortality risk. Further research to assess the adverse effects of type 2 DM on long-term survival may be warranted.

## Introduction

As the world population ages, surgical cases volume increases gradually. Postoperative ischemic stroke is a rare, under-recognized, and life-threatening neurological complication of surgery with high mortality and disability (Wong et al., 2000; Vlisides and Mashour, 2016). Compared to community-onset stroke, patients who are experiencing postoperative ischemic stroke have worse outcomes after surgery (Selim, 2007; Saltman et al., 2015). With a delay to diagnostic imaging, a narrow time window, and the high risk of bleeding, less than 5% of eligible patients benefit from thrombolysis and the majority may suffer from poor prognosis (Saltman et al., 2015; Thiebaut et al., 2018).

The presence of type 2 diabetes mellitus (DM), which affected 11.2% of adults in China, has been critically linked to increased mortality (Li et al., 2020). Meanwhile, type 2 diabetes is characterized by impaired insulin signaling, hyperglycemia, lipid metabolism dysfunction, and defective glucagon secretion (Feldman et al., 2017, 2019). Altered insulin signaling provokes apoptosis by inhibiting axonal growth and neurotrophin secretion (Pop-Busui et al., 2017; Calcutt, 2020). Abnormal glucose metabolism results in hypoxia and microcirculation dysfunction, which in turn leads to an irreversible impairment of neurons, astrocytes, and endothelial cells (Sloan et al., 2021).

Given the high prevalence and adverse effects of type 2 DM, we hypothesized that the potential risk of long-term survival outcomes attributable to type 2 DM could be substantial. Herein, we conducted a retrospective study to examine the association between type 2 DM and long-term survival outcomes in the patients with postoperative ischemic stroke.

## Research Design and Methods

This retrospective cohort study was approved by the Medical Ethics Committee of Chinese PLA General Hospital (reference number: S2021-493-01), and the requirement for informed content was waived. The manuscript of the cohort study adheres to the Strengthening the Reporting of Observational Studies in Epidemiology (STROBE) guidelines (Supplementary Table 1).

### Study Subjects

**FIGURE 1:**
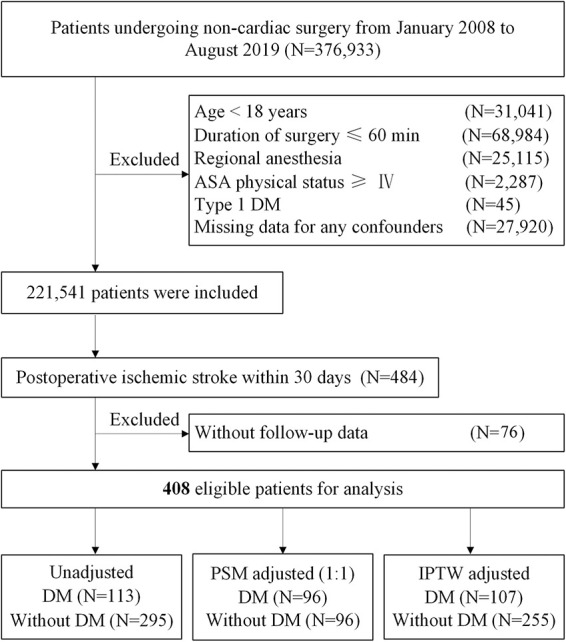
Study flow diagram. ASA, American Society of Anesthesiologists; DM, diabetes mellitus; PSM, propensity score matching; IPTW, inverse probability treatment weighting.

We identified the patients who had undergone non-cardiac surgery between January 1, 2008, and August 31, 2019, at Chinese PLA General Hospital, a tertiary academic hospital in Beijing, China. We excluded patients with (1) age < 18 years, (2) duration of surgery ≤ 60 min, (3) regional anesthesia, (4) American Society of Anesthesiologists (ASA) physical status ≥ IV, (5) type 1 DM, and (6) missing data for any confounders. For patients who underwent multiple surgeries within the study period, only the first eligible surgery was included. Additionally, we screened the patients who were diagnosed to have a postoperative ischemic stroke within 30 days after surgery, identified through ICD 9/ICD 10 diagnosis codes (Supplementary Table 2). If patients were lost to follow-up, the patients were excluded. The screening process of eligible patients is depicted in Figure 1.

### Study Outcome

The outcome of interest was overall survival (OS). The survival data after surgery were derived from hospital medical records, follow-up database, and confirmed by the Chinese Center for Disease Control and Prevention (CCDC). OS was defined as the period from the date of surgery to the day when the death occurred after surgery or the remainder were censored at the end of the follow-up point (June 30, 2021) (Rich et al., 2010).

### Exposure of Interest and Covariates

The exposure of interest was type 2 DM (hereafter simplified as DM) before the postoperative ischemic stroke event, identified through ICD 9/ICD 10 diagnosis codes (Supplementary Table 2).

Covariates of interest included age, sex, body mass index (BMI), American Society of Anesthesiologists physical status, other comorbidities (hypertension, previous ischemic stroke, myocardial infarction, peripheral vascular disease, and chronic kidney disease), use of preoperative medications (β blockers, aspirin), preoperative serological examination and index (hemoglobin, albumin, total bilirubin, prothrombin time, neutrophil-lymphocyte ratio, and platelet to lymphocyte ratio), surgery-related data [emergency surgery, surgery type, malignant tumor, duration of surgery, estimated blood loss, preoperative mean arterial pressure (MAP), blood products depot, colloids infusion, crystalloids infusion, non-steroid anti-inflammatory drugs (NSAIDs), morphine equivalents, and ICU admission after surgery], stroke severity (National Institutes of Health Stroke Scale), stroke laterality, stroke location, large hemispheric infarction (LHI), and thrombolysis.

Postoperative ischemic stroke is defined as a brain infarction of ischemic etiology with motor, sensory, or cognitive dysfunction (e.g., hemiplegia, hemiparesis, aphasia, sensory deficit, and impaired memory) 30 days after surgery (Mashour et al., 2011; Sacco et al., 2013; Vlisides and Moore, 2021). Diagnoses of stroke are confirmed by a combination of neuroimaging and clinical evidence of cerebrovascular ischemia during hospital stay. Preoperative MAP was determined on the first blood pressure in the operation room. Stroke laterality, stroke location, and LHI were suggested by CT or MRI. LHI was defined as follow: (1) suffered from >1/3 infarction of the middle cerebral artery (MCA) territory within 6 h after the onset of stroke (or >1/2 MCA territory from 6 h to 7 days after onset) by the assessment of CT scan, (2) exceeded 80 ml within 6 h after the onset of stroke (or 145 ml within 14 h after onset) for infarction volume by appraisal of the MRI), and (3) suffered from >3 cm infarction of cerebellar region on the MRI (Sheth et al., 2016; Vorasayan et al., 2019).

### Statistical Analysis

Baseline characteristics and outcomes of the patients were summarized through frequencies and descriptive statistics. Continuous variables are expressed as mean (SD), or median (interquartile range, IQR), and categorical variables are presented as *n* (%). Kaplan-Meier method was performed to compare OS between groups.

To control the potential confounding effects, we performed multivariate Cox regression analysis with step-wise selection to evaluate the relationship between DM and OS. In Cox regression analysis, multiple models were conducted with different covariates to calculate the hazard ratio (HR) of OS and DM (Table 2).

In sensitivity analyses, we conducted propensity score (PS) analysis, such as propensity score matching (PSM) and inverse probability treatment weighting (IPTW) to examine the OS associated with DM (Haukoos and Lewis, 2015; Grool et al., 2016). For adjusting between-group differences, PSs were developed to reflect the probability of each patient having DM (Kurth et al., 2006; Austin, 2011). PS, a composite score, was derived from synthesized baseline characteristics (Williamson and Forbes, 2014). Clinically relevant covariates (the aforementioned 34 covariates) were included in the multivariate logistic regression model to yield a PS (Zhang et al., 2020). In PSM, matching between two groups was randomly conducted with PS at a 1:1 ratio, by using the greedy nearest-neighbor approach, with a caliper width of the PS with 0.1 pooled SD (Rosenbaum and Rubin, 2012). In IPTW, symmetric trimming was performed to minimize the adverse effects of extreme PS outliers. We excluded the patients whose estimated PS was beyond the range (10–90%) (Li et al., 2019). After obtaining matched data or weighted data, Kernel density plots and standardized mean difference (SMD) were applied to assess the balance of covariates between the two groups. An SMD < 0.2 was deemed as acceptable deviations for the particular covariate (Brookhart et al., 2006; Cheung et al., 2019). The association between DM and OS was estimated using multivariate Cox regression analysis to calculate the adjusted HR.

Advanced age, malignant tumor surgery, LHI, HbA_1c_ level, and the follow-up time were associated with shortened OS in several previous meta-analyses or studies (Sheth et al., 2018; GBD 2019 Diseases and Injuries Collaborators, 2020; Wang et al., 2021). Thus, we performed subgroup analysis according to age, malignant tumor surgery, LHI, HbA_1c_ level, and the follow-up time to explore the potential interaction. Multivariate Cox regression analyses were separately performed on each individual subgroup to calculate the adjusted HR.

A two-sided *p* < 0.05 was considered statistically significant. Statistical analyses were performed using IBM SPSS Statistics (version 26; IBM Corp) and R program (version 4.0.5; R Foundation for Statistical Computing, Vienna, Austria), along with Table 1, MatchIt, car, survival, survminer, survey, glm 2, and ggplot 2 packages.

**TABLE 1:** Baseline characteristics unadjusted sample, propensity score-matched sample, and inverse probability of treatment-weighted sample.

Characteristic	Unadjusted sample (*N* = 408)			PSM adjusted (1:1) (*n* = 192)			IPTW adjusted (*n* = 362)*		
	Patients without DM (*n* = 295)	Patients with DM (*n* = 113)	SMD	Patients without DM (*n* = 96)	Patients with DM (*n* = 96)	SMD	Patients without DM (*n* = 255)	Patients with DM (*n* = 107)	SMD
Age, years^†^	64.0 (54.0, 70.0)	65.0 (59.0, 71.0)	0.314	67.0 (59.0, 73.0)	65.0 (58.0, 70.0)	0.007	66.0 (58.0, 72.0)	65.0 (58.0, 70.0)	0.068
Female sex (%)^†^	133 (45.1)	50 (44.2)	0.017	45 (46.9)	44 (45.8)	0.021	163.5 (44.7)	150.3 (44.2)	0.011
BMI, kg/m^2†^	24.5 (22.5, 26.8)	25.4 (23.0, 27.6)	0.296	25.3 (22.8, 27.3)	25.2 (22.8, 27.4)	0.065	24.9 (22.8, 27.2)	25.1 (22.3, 27.2)	0.007
**ASA physical status (%)^†^**									
Class I	11 (3.7)	2 (1.8)	0.205	2 (2.1)	2 (2.1)	0.022	7.8 (2.1)	7.7 (2.3)	0.082
Class II	199 (67.5)	69 (61.0)		57 (59.4)	58 (60.4)		236.4 (64.6)	206.1 (60.6)	
Class III	85 (28.8)	42 (37.2)		37 (38.5)	36 (37.5)		121.8 (33.3)	126.2 (37.1)	
Hypertension (%)^†^	139 (47.1)	71 (62.8)	0.320	56 (58.3)	57 (59.4)	0.021	205.2 (56.1)	191.9 (56.5)	0.008
Previous ischemic stroke (%)^†^	74 (25.1)	51 (45.1)	0.430	41 (42.7)	40 (41.7)	0.021	124.9 (34.1)	121.6 (35.7)	0.033
Myocardial infarction (%)^†^	17 (5.8)	15 (13.3)	0.258	10 (10.4)	13 (13.5)	0.096	31.5 (8.6)	31.8 (9.3)	0.022
Peripheral vascular disease (%)^†^	57 (19.3)	27 (23.9)	0.111	24 (25.0)	20 (20.8)	0.099	83.6 (22.8)	74.2 (21.8)	0.027
Chronic kidney disease (%)^†^	7 (2.4)	4 (3.5)	0.069	2 (2.1)	1 (1.0)	0.084	8.3 (2.2)	6.4 (1.9)	0.025
Preoperative β blockers (%)^†^	34 (11.5)	14 (12.4)	0.027	13 (13.5)	11 (11.5)	0.098	48.6 (13.3)	34.2 (10.0)	0.098
Preoperative aspirin (%)^†^	83 (28.1)	46 (40.7)	0.267	33 (34.4)	37 (38.5)	0.087	121.5 (33.2)	127.1 (37.4)	0.090
Preoperative Hb, g/L^†^	132.0 (122.0, 145.5)	132.0 (119.0, 145.0)	0.100	131.0 (119.0, 141.0)	132.5 (120.0, 146.3)	0.089	132.0 (122.0, 144.7)	131.7 (118.4, 142.0)	0.092
Preoperative ALB, g/L^†^	40.5 (37.8, 42.6)	40.5 (38.2, 43.0)	0.033	40.1 (37.3, 42.5)	40.5 (38.3, 43.3)	0.078	40.4 (37.5, 42.6)	40.5 (37.8, 43.1)	0.049
Preoperative TBIL, μmol/L^†^	10.8 (8.10, 14.7)	10.0 (7.2, 13.7)	0.035	10.9 (8.0, 14.9)	10.1 (7.1, 13.7)	0.020	10.5 (8.0, 14.5)	9.8 (7.0, 14.2)	0.032
Preoperative PT, s^†^	13.1 (11.5, 14.5)	13.6 (11.4, 14.9)	0.064	13.2 (11.5, 14.8)	13.8 (11.4, 15.1)	0.062	13.1 (11.3, 15.0)	13.3 (11.6, 14.4)	0.053
Preoperative NLR^†^	2.3 (1.7, 3.9)	2.3 (1.7, 3.5)	0.079	2.2 (1.6, 3.8)	2.2 (1.7, 3.3)	0.036	2.3 (1.7, 3.8)	2.3 (1.7, 3.9)	0.020
Preoperative PLR^†^	132.8 (96.6, 174.1)	130.2 (99.1, 176.0)	0.012	125.3 (102.2, 173.6)	124.6 (94.1, 168.8)	0.075	129.4 (94.9, 169.5)	126.1 (94.0, 176.0)	0.064
Emergency (%)^†^	34 (11.5)	10 (8.8)	0.089	8 (8.3)	9 (9.4)	0.017	35.6 (9.7)	34.9 (10.3)	0.018
**Surgery type (%)^†^**									
Spine	29 (9.8)	19 (16.8)	0.499	13 (13.5)	16 (16.7)	0.084	47.2 (12.9)	44.0 (12.9)	0.083
Intra-abdominal surgery	41 (13.9)	31 (27.4)		24 (25.0)	25 (26.0)		73.1 (20.0)	71.9 (21.1)	
Joint arthroplary	25 (8.5)	9 (8.0)		4 (4.2)	7 (7.3)		33.6 (9.2)	32.9 (9.7)	
Oral and maxillofacial	13 (4.4)	4 (3.5)		4 (4.2)	3 (3.1)		13.4 (3.7)	18.9 (5.6)	
Urologic	22 (7.5)	10 (8.8)		10 (10.4)	9 (9.4)		32.8 (9.0)	31.4 (9.2)	
Neurosurgery	112 (38.0)	28 (24.9)		27 (28.1)	25 (26.0)		117.1 (31.9)	98.8 (29.1)	
Thoracic or vascular	11 (3.7)	4 (3.5)		3 (3.1)	4 (4.2)		15.3 (4.2)	9.7 (2.9)	
Other (ENT, etc.)	42 (14.2)	8 (7.1)		11 (11.5)	7 (7.3)		33.5 (9.1)	32.4 (9.5)	
Malignant tumor surgery (%)^†^	109 (36.9)	55 (48.7)	0.239	46 (47.9)	45 (46.9)	0.021	156.7 (42.8)	151.9 (44.7)	0.038
Duration of surgery, min^†^	202.0 (136.0, 296.5)	190.0 (141.0, 265.0)	0.144	201.0 (149.8, 266.3)	190.0 (140.0, 266.8)	0.076	205.0 (139.6, 295.0)	211.8 (143.2, 288.6)	0.022
Estimated blood loss, mL^†^	200.0 (50.0, 300.0)	200.0 (100.0, 300.0)	0.042	200.0 (80.0, 300.0)	175.0 (100.0, 300.0)	0.003	200.0 (68.9, 400.0)	200.0 (100.0, 300.0)	0.048
Preoperative MAP, mmHg^†^	96.7 (90.0, 106.3)	99.3 (92.0, 105.7)	0.090	97.7 (92.3, 103.3)	98.2 (91.3, 104.5)	0.061	97.7 (92.1, 106.7)	97.6 (91.0, 104.8)	0.075
Blood products depot (%)^†^	58 (19.7)	24 (21.2)	0.039	16 (16.7)	18 (18.8)	0.092	70.9 (19.4)	79.2 (23.3)	0.097
Colloids infusion, ml/kg/min^†^	0.04 (0.03, 0.07)	0.05 (0.02, 0.06)	0.146	0.05 (0.03, 0.07)	0.05 (0.02, 0.07)	0.075	0.05 (0.03, 0.07)	0.05 (0.02, 0.06)	0.086
Crystalloids infusion, ml/kg/min^†^	0.12 (0.09, 0.16)	0.13 (0.10, 0.18)	0.144	0.12 (0.09, 0.14)	0.13 (0.10, 0.16)	0.091	0.12 (0.10, 0.16)	0.12 (0.10, 0.17)	0.090
NSAIDs (%)^†^	223 (75.6)	91 (80.5)	0.120	77 (80.2)	76 (79.2)	0.026	284.9 (77.9)	259.7 (76.4)	0.031
Morphine equivalents, mg^†‡^	135.0 (105.0, 165.0)	150.0 (120.0, 180.0)	0.146	150.0 (120.0, 165.0)	150.0 (120.0, 180.0)	0.069	145.0 (105.0, 165.0)	150.0 (120.0, 180.0)	0.093
ICU admission after surgery (%)^†^	144 (48.8)	52 (46.0)	0.129	43 (44.8)	45 (46.9)	0.042	170.6 (46.6)	159.1 (46.8)	0.006
Stroke severity (NIHSS) ^†^	12 (7,18)	13 (5,21)	0.094	12 (8,15)	13 (5,16)	0.020	13.0 (6.0, 18.0)	13.0 (6.0, 20.0)	0.059
**Stroke laterality (%)^†^**									
Left	107 (36.3)	47 (41.6)	0.140	36 (37.5)	37 (38.5)	0.053	125.8 (34.4)	133.8 (39.3)	0.117
Right	113 (38.3)	36 (31.9)		38 (39.6)	35 (36.5)		143.3 (39.2)	124.9 (36.7)	
Bilateral	75 (25.4)	30 (26.5)		22 (22.9)	24 (25.0)		96.8 (26.4)	81.7 (24.0)	
**Stroke location (%)^†^**									
Cortical	60 (20.3)	12 (10.6)	0.332	12 (12.5)	10 (10.4)	0.089	52.6 (14.4)	38.3 (11.3)	0.096
Subcortical	144 (48.8)	63 (55.8)		57 (59.5)	56 (58.3)		199.2 (54.4)	192.3 (56.5)	
Cerebellar	4 (1.4)	2 (1.8)		1 (1.0)	2 (2.1)		6.5 (1.8)	7.2 (2.1)	
Brainstem	1 (0.3)	3 (2.7)		1 (1.0)	0 (0.0)		3.8 (1.0)	3.2 (0.9)	
Multiple	86 (29.2)	33 (29.2)		25 (26.0)	28 (29.2)		103.9 (28.4)	99.0 (29.2)	
LHI (%)^†^	45 (15.3)	12 (10.6)	0.138	9 (9.4)	7 (7.3)	0.092	50.1 (13.7)	41.8 (12.3)	0.096
Thrombolysis (%)^†^	1 (0.3)	0 (0.0)	0.006	0 (0.0)	0 (0.0)	<0.001	0 (0.0)	0 (0.0)	<0.001
Follow-up, months	50.9 (24.8, 89.1)	33.8 (17.0, 67.8)	0.217	54.6 (29.7, 94.1)	35.7 (17.8, 72.8)	0.259	50.6 (27.5, 88.0)	30.9 (17.0, 72.2)	0.381
Midian OS (95% CI), months	52.8 (47.2, 60.2)	34.3 (29.4, 40.9)	0.318	55.9 (47.2, 68.2)	36.6 (31.4, 49.6)	0.352	51.8 (47.0, 59.4)	31.7 (25.4, 41.4)	0.452

*The data are shown as the median (interquartile range), n (%), or mean ± SD.*

*Proportions and medians are weighted using IPTW.

*^†^Variables included in the propensity score.*

*^‡^Including those intraoperatively and postoperatively (up to 7 days after surgery). Morphine 30 mg (per os) = morphine 10 mg (iv) = sufentanil 10 μg (iv) = fentanyl 100 μg (iv) = remifentanil 100 μg (iv) = 100 mg tramadol (iv) = tramadol 200 mg (per os) = oxycodone 15 mg (per os) = dezocine 10 mg (iv) = pethidine 100 mg (iv).*

*DM, diabetes mellitus; PSM, propensity score matching; IPTW, inverse probability of treatment weighting; SMD, standardized mean difference; BMI, body mass index; ASA, American Society of Anesthesiologists; Hb, hemoglobin; ALB, albumin; TBIL, total bilirubin; PT, prothrombin time; NLR, neutrophil-lymphocyte ratio; PLR, platelet to lymphocyte ratio; ENT, ear, nose, and throat; MAP, mean arterial pressure; NSAIDs, non-steroid anti-inflammatory drugs; ICU, intensive care unit; NIHSS, national institutes of health stroke scale; LHI, large hemispheric infarction; OS, overall survival; CI, confidence interval.*

## Results

### Baseline Characteristics of Patients

From January 1, 2008, to August 31, 2019, at Chinese PLA General Hospital, a total of 2,21,541 patients who underwent non-cardiac surgery were included, of whom 484 (0.22%) patients were diagnosed to have an ischemic stroke within 30 days after surgery. After excluding 76 patients without follow-up data, 408 of 484 (84.3%) eligible patients with postoperative ischemic stroke remained in the cohort, of whom 113 (27.7%) had DM (Figure 1). During a median follow-up of 46.2 months (IQR: 21.1, 84.2), the overall all-cause mortality was 49.0% (200/408).

Baseline characteristics of the postoperative ischemic stroke patients with or without DM are summarized in Table 1. Some of the patient characteristics were similar between the two groups. However, several characteristics, such as BMI, medical history, preoperative aspirin, surgery type, malignant tumor, and stroke location, differed significantly between the two groups. Postoperative ischemic stroke patients with the DM group had more cardiovascular comorbidities (hypertension, myocardial infarction, previous ischemic stroke, and peripheral vascular disease) and higher long-term use of aspirin, than did those without DM. In terms of surgery type, the patients with the DM group underwent more spine or intra-abdominal surgery, whereas the patients without the DM group had more neurosurgery. Compared with patients without the DM group, patients with the DM group had higher BMI and more malignant tumor surgery.

After adjustment with PSM or IPTW method, most of the covariates were well balanced, with the standardized mean difference less than 0.10 for all covariates (Table 1).

### Causes of Death

In total, 200 patients with postoperative ischemic stroke had died during the follow-up period. In the analysis of causes of death, nine patients without specific causes of death were excluded. More than half of the postoperative ischemic stroke patients had subsequently died from cerebrovascular diseases and cardiovascular diseases. More patients with postoperative ischemic stroke had died from cerebrovascular diseases (35.1%) than from heart disease (22.5%). Meanwhile, the mortality rate of postoperative ischemic stroke patients with DM due to all cerebrovascular diseases was higher than that in those without DM (44.9 vs. 29.5%, DM vs. no DM). However, the mortality rate of postoperative ischemic stroke patients without DM due to cancer was much higher than that in those with DM (18.8 vs. 30.3%, DM vs. no DM) (Supplementary Table 3).

### Primary Analysis

**FIGURE 2:**
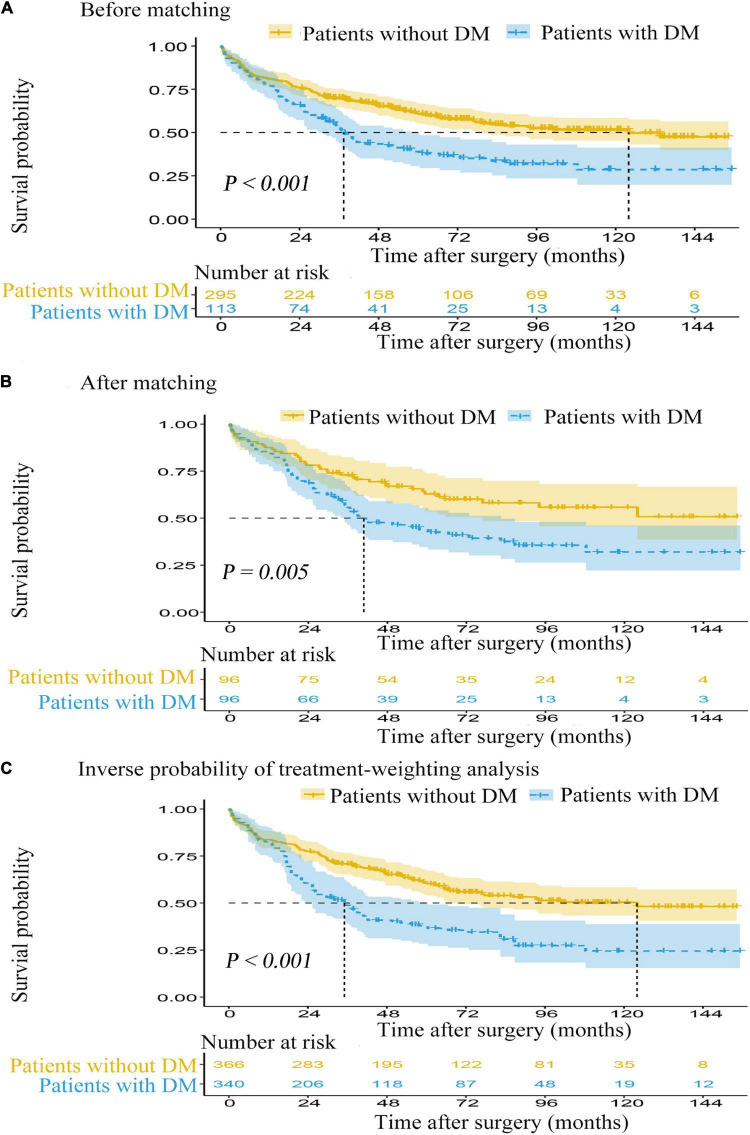
The Kaplan-Meier survival curves for overall survival from the date of surgery. **(A)** Before matching. **(B)** After matching. **(C)** Inverse probability of treatment-weighting analysis. DM, diabetes mellitus.

Median follow-up time for postoperative ischemic stroke patients with DM was 33.8 months (IQR: 17.0–67.8) and 50.9 months (IQR: 24.8–89.1) for those without DM. In the Kaplan-Meier survival curves for OS, the postoperative ischemic stroke patients with DM had a significantly unfavorable survival (log-rank test, *p* < 0.001). The median OS time and OS rates at 3, 5, and 10 years were significantly lower for the postoperative ischemic stroke patients with DM than those without DM [median OS (95% CI): 34.3 (29.4, 40.9) vs. 52.8 (47.2, 60.2) months, *p* < 0.001; 3 years OS: 52.2% (59/113) vs. 69.5% (205/295), *p* < 0.001; 5 years OS: 41.6% (47/113) vs. 62.4% (184/295), *p* < 0.001; 10 years OS: 37.2% (42/113) vs. 56.6% (167/295), *p* < 0.001; Figure 2A].

In the Cox proportional hazards model regression analysis (Table 2), the presence of DM was significantly associated with a shortened OS in both the univariate analysis (HR: 1.763; 95% CI: 1.319–2.356; *p* < 0.001) and the multivariable Cox regression adjustment (HR range: 1.775–2.039; *p* < 0.001 for all). The detailed data of univariate and multivariable Cox regression analyses for OS are displayed in Supplementary Table 4.

**TABLE 2:** Association between diabetes mellitus (DM) and overall survival (OS) using the Cox proportional hazards regression model and propensity score analysis.

Analysis method	HR	95% CI	*P* value
**Cox proportional hazards model regression analysis (*n* = 408)**			
Model 1 (univarible analysis)*	1.763	1.319–2.356	<*0.001*
Model 2 (preoperative patient- related covariates adjusted)^†^	2.039	1.475–2.818	<*0.001*
Model 3 (surgery-related covariates adjusted)^‡^	1.775	1.310–2.406	<*0.001*
Model 4 (postoperative patient- related covariates adjusted)^§^	2.061	1.520–2.792	<*0.001*
Model 5 (fully adjusted)^| |^	1.947	1.397–2.713	<*0.001*
**Propensity score analysis (multivarible analysis)**			
PS matching (*n* = 192)^¶^	2.19	1.354–3.540	0.001
IPTW (*n* = 362)^#^	2.551	1.769–3.679	<*0.001*

*DM, diabetes mellitus; OS, overall survival; HR, hazard ratio; CI, confidence interval; PS, propensity score; IPTW, inverse probability of treatment weighting.*

*Model 1 was a univariate crude model.

*^†^Model 2 included age, female sex, BMI, ASA physical status, hypertension, previous ischemic stroke, myocardial infarction, peripheral vascular disease, chronic kidney disease, preoperative β blockers, preoperative aspirin, preoperative Hb, preoperative ALB, preoperative TBIL, preoperative PT, preoperative NLR, preoperative PLR.*

*^‡^Model 3 included emergency surgery, surgery type, malignant tumor, duration of surgery, estimated blood loss, preoperative MAP, blood products depot, colloids infusion, crystalloids infusion, NSAIDs, morphine equivalents, ICU admission after surgery.*

*^§^ Model 4 included stroke severity, stroke laterality, stroke location, large hemispheric infarction, and thrombolysis.*

*^| |^ Model 5 included all the confounders. Univariate and multivariable results are displayed in Supplementary Table 4.*

*^¶^ 96 Pairs were matched by propensity score. Univariate and multivariable results are displayed in Supplementary Table 5.*

*^#^In IPTW, we excluded the patients whose estimated propensity score was beyond the range (10–90%), thus there were 362 patients remaining. Univariate and multivariable results are displayed in Supplementary Table 6.*

### Propensity Score-Matched Analysis and Adjustment

**FIGURE 3:**
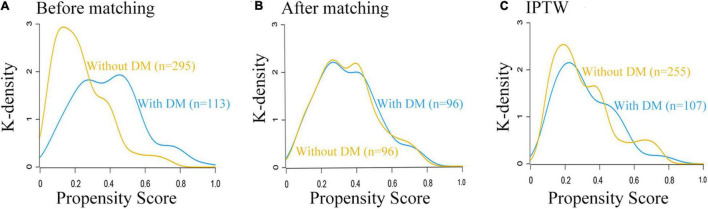
Distribution of propensity scores in the postoperative ischemic stroke patients with DM group and without DM group. **(A)** Before matching. **(B)** After matching. **(C)** IPTW. DM, diabetes mellitus.

Prior to PS adjustment, median PS in postoperative ischemic stroke patients with DM group was 0.399 (IQR: 0.246–0.501) vs. 0.202 (IQR: 0.118–0.325) in those without DM group. After matching, 96 patients remained in postoperative ischemic stroke patients with DM group and 96 patients remained in those without DM group. The distribution of PSs among the two groups is graphically displayed by kernel density estimation before and after adjustment (Figures 3A,B). In the post-matched cohort, the mean (SD) PS was similar between those reporting DM [0.350 (0.167)] and those patients without DM [0.347 (0.165)], and all confounders were well balanced between the two groups (standardized mean difference less than 0.1; Table 1). The Kaplan-Meier survival plot suggests that the presence of DM was still significantly associated with a shortened OS (log-rank test, *p* = 0.005; Figure 2B). In the multivariable Cox model after PSM (*n* = 96), the association between the presence of DM and OS yielded robust results (HR: 2.190; 95% CI: 1.354–3.540; *p* = 0.001; Table 2 and Supplementary Table 5).

### Inverse Probability Treatment Weighting Analysis and Adjustment

A total of 362 patients with postoperative ischemic stroke remained after the IPTW trimming, of whom 107 (29.6%) patients in those were with the DM group and 255 (70.4%) patients in those were without the DM group. After IPTW adjustment, the weighted distribution of PSs among the two groups is shown in Figure 3C. The mean (SD) weight was 1.950 (1.316) with a range from 1.091 to 10.333. In the IPTW cohort, all covariates were between-group balanced (SMD < 0.1), except for stroke laterality (SMD: 0.117) (Table 1). In the Kaplan-Meier curves or IPTW Cox regression analysis, the OS also remained significantly unfavorable in the postoperative ischemic stroke patients with DM group compared with those without DM group [log-rank test, *p* < 0.001; HR (95% CI): 2.551 (1.769, 3.679), *p* < 0.001; Figure 2C, Table 2 and Supplementary Table 6].

### Subgroup Analysis

**FIGURE 4:**
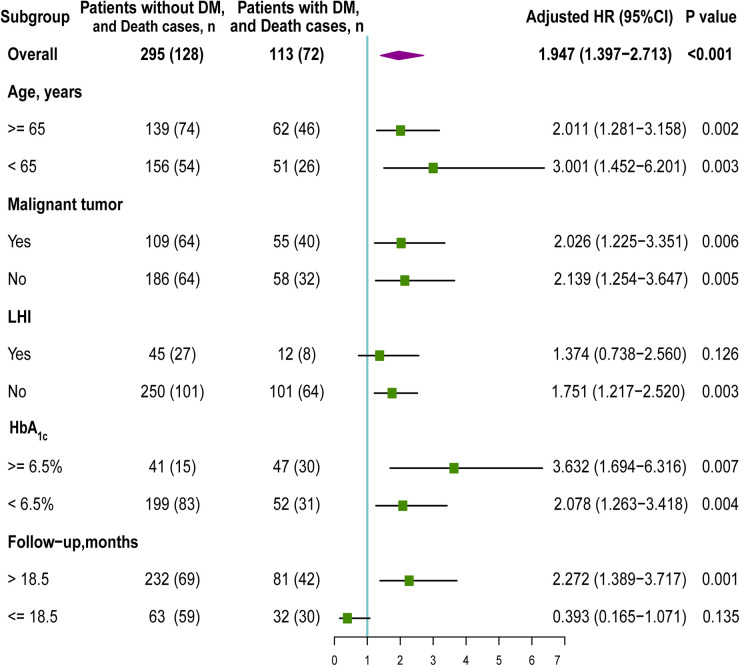
Subgroup analysis of the association between DM and overall survival (OS). HR, hazard ratio; DM, diabetes mellitus; LHI, large hemispheric infarction.

Among 113 postoperative ischemic stroke patients with DM, 62 (54.9%) patients were aged ≥ 65 years, 55 (48.7%) underwent malignant tumor surgery, 12 (10.6%) presented with LHI, and 81 (71.7%) were with the follow-up time > 18.5 months. Figure 4 shows the subgroup analysis according to age, malignant tumor, LHI, HbA1c level, and follow-up. The HR of the presence of DM was significant in the age subgroup [≥65 years: HR (95% CI): 2.011 (1.281, 3.158), *p* = 0.002; <65 years: HR (95% CI): 3.001 (1.452, 6.201), *p* = 0.003]. Additionally, the increased risk of unfavorable OS was noted among those who underwent malignant tumor surgery (HR: 2.026; 95% CI: 1.225–3.351; *p* = 0.006) and those who did not undergo malignant tumor surgery (HR: 2.139; 95% CI: 1.254–3.647; *p* = 0.005). The presence of DM was only significantly associated with increasing all-cause death in negative LHI group (HR: 1.751; 95% CI: 1.217–2.520; *p* = 0.003), whereas it was not significant in positive LHI group (HR: 1.374; 95% CI: 0.738–2.560; *p* = 0.126). The association between DM and OS was only significant for follow-up time > 18.5 months subgroup [HR (95% CI): 2.272 (1.389, 3.717), *p* = 0.001, DM vs. no DM], whereas it was not significant for follow-up time ≤ 18.5 months subgroup [HR (95% CI): 0.393 (0.165, 1,071), *p* = 0.135, DM vs. no DM). Since 69 patients without HbA1c data were excluded, we performed HbA_1c_ level subgroup analyses of 339 patients with HbA_1c_ data. Suboptimal diabetes control (HbA_1c_ ≥ 6.5%) was associated with a higher risk of unfavorable survival [HR (95% CI): 3.632 (1.694, 6.316), *p* = 0.007] than those with HbA_1c_ < 6.5% [HR (95% CI): 2.078 (1.263, 3.418), *p* = 0.004].

## Discussion

In this retrospective cohort study of 408 patients with postoperative ischemic stroke, we showed that type 2 diabetes was associated with a worsened prognosis for OS. After several statistical adjusting approaches for the difference between postoperative ischemic stroke patients with DM and those patients without DM, this finding remained consistent.

Individually, evidence about the increased risk for cardiovascular disease, neuropathy, and mortality with DM or stroke are unequivocal (Hu and Jia, 2018; Meagher et al., 2018). Patients with DM were reported of 2–4 times increased risk in death and cardiovascular events as great as the patients without DM, particularly, in the elderly population (Chen et al., 2020). According to the works of literature, a range of interventions that target elevated levels of glycated hemoglobin, high blood pressure, and elevated cholesterol have proved effective in reducing the risks of complications of DM (Rawshani et al., 2018). Stroke in the community is independently associated with an adjusted increase in physical disability, cognitive dysfunction, and mortality. When stroke in the community co-occurs with DM, the potential synergies will have multiplicative mortality risk (Emerging Risk Factors Collaboration et al., 2015). Additionally, postoperative ischemic stroke also shows prolonged hospital stays, increased risks of 30-days mortality and disability, and poor prognosis for long-term survival (Selim, 2007). Compared with DM and stroke in the community in which the latest insights with respect to an unfavorable prognosis have reached a consensus, the field of combination of postoperative ischemic stroke and DM lags behind.

The major hurdle of the postoperative ischemic stroke study is the lower incidence, requiring a huge sample size. In the POISE trial of 8,351 patients who underwent non-cardiac surgery, 60 (0.7%) patients developed stroke within 30 days after surgery (POISE Study Group et al., 2008). A recent retrospective cohort study, such as 1,165,750 surgical patients, suggests that postoperative ischemic stroke was found in 2,500 patients (0.25%) (Woo et al., 2021). In the current study, 484 patients with postoperative ischemic stroke were found in a large cohort of 2,21,541 patients with non-cardiac surgery. Compared to aforementioned studies reporting, the stroke rate (0.22%) was comparable with an incidence of stroke between 0.1 and 0.7% (Vasivej et al., 2016). When postoperative ischemic stroke co-occurred with DM, more patients were died from cerebrovascular diseases due to the potential synergies between these two factors. Multicenter, well-designed, and prospective trials are welcomed to further verify our hypothesis. Based on the cohort of patients with postoperative ischemic stroke, we demonstrated the association between DM and OS. Furthermore, we noticed that the subgroups still rendered the finding statistically significant with advanced age and malignant tumor surgery, which indicated a robustly adverse effect on the long-term survival of DM in the patients. However, the adverse effect of DM only existed in the negative LHI group, whereas it remained insignificant in the positive LHI group. LHI is a devastating stroke affecting the majority or complete occlusion of MCA and usually accompanies MCE (Liebeskind et al., 2019). In the current study, 57 (14.0%) patients with postoperative ischemic stroke become LHI; the rate was approximate to prior studies reporting the incidence of LHI less than 10% (Wijdicks et al., 2014). LHI showed a higher mortality (61.4 vs. 47.0%, *p* < 0.001, LHI vs. no LHI) and a shorten OS [median OS (95% CI): 31.6 (26.5, 37.1) vs. 48.4 (43.2, 55.2) months, *p* < 0.001, LHI vs. no LHI]. Therefore, we speculated that the association between DM and OS was eliminated due to a faster progression and high severity of LHI. The subgroup analysis of HbA_1c_ level in this study showed that postoperative ischemic stroke with DM with HbA1c ≥ 6.5% had a much higher risk of death, implying that strict glycemic control might help to improve survival.

This study has the following merits. First, as the postoperative ischemic stroke with lower incidence, we constructed a larger retrospective database with 3,76,933 surgical patients to screen the eligible patients with postoperative ischemic stroke as much as possible. Second, the survival data after surgery were derived from hospital medical records and a follow-up database. We validated the precision of survival data from the Chinese CCDC. Third, we made efforts to integrate preoperative, intraoperative, and postoperative data into this retrospective database. Thus, we included a variety of potential confounding factors, such as preoperative, intraoperative, and postoperative data, which allow for precise effect size evaluation. Fourth, we also assessed stroke laterality, stroke location, and especially LHI by CT or MRI imaging to reflect the difference in stroke status. Fifth, sensitivity analyses, such as PSM or IPTW analysis and subgroups analysis, were successfully applied to further validate the robustness of our findings. Last, to the best of our knowledge, this is the first study to evaluate the association between DM and long-term OS for patients with ischemic stroke after surgery.

Several potential limitations in our study deserve closer attention. First, we cannot draw conclusions regarding cause-effect relationships due to this retrospective study. Therefore, this causality between DM and long-term OS for patients with postoperative ischemic stroke will need to be validated with larger randomized clinical trials. Second, the results derived from a single-center study and thus the generalizability of our findings may be limited in other centers. Thus, multicenter, well-designed, and prospective trials are welcomed to further verify our hypothesis. Third, residual and unmeasured potential confounding cannot be completely ruled out in an observational study. Fourth, given the high specificity but low sensitivity of ICD codes, the true incidence may be underestimated with DM or peripheral vascular disease. Fifth, due to insufficient awareness of the HbA_1c_ test among the surgeons, it is not a routine test for screening diabetes between the years 2008 and 2010 in our center. Thus, we did not have enough HbA_1c_ data to evaluate overall glycemic control before the surgery. In the study, we only performed the HbA_1c_ subgroup analyses of 339 patients with HbA_1c_ data. Sixth, despite many efforts in obtaining more relevant data, we still lacked the preoperative data of antidiabetic medications, such as insulin and metformin, and long-term data of glycemic control. These potential confounders should be adjusted.

## Conclusion

Type 2 DM was associated with a unfavorable prognosis for OS in patients with postoperative ischemic stroke. When postoperative ischemic stroke co-occurred with Type 2 DM, the potential synergies would have multiplicative mortality risk. Future larger randomized clinical trials are required to confirm the adverse effects of type 2 DM on long-term survival.

## Data Availability Statement

The original contributions presented in the study are included in the article/Supplementary Material, further inquiries can be directed to the corresponding authors.

## Ethics Statement

The studies involving human participants were reviewed and approved by the Medical Ethics Committee of Chinese PLA General Hospital. Written informed consent for participation was not required for this study in accordance with the national legislation and the institutional requirements.

## Author Contributions

WM, LW, and MN conceived and designed the study. FZ, MN, YM, YY, and MS contributed to the data extraction and acquisition. FZ, MN, and LW drafted the manuscript. YM, HL, and YL analyzed and interpreted the data. JL and JC supervised the study. FZ, MN, YM, WM, and LW critically revised the manuscript for important intellectual content. WM was the guarantor of this study and, as such, had full access to all the data in the study and takes responsibility for the integrity of the data and the accuracy of the data analysis. All authors contributed to the article and approved the submitted version.

## Conflict of Interest

The authors declare that the research was conducted in the absence of any commercial or financial relationships that could be construed as a potential conflict of interest.

## Publisher’s Note

All claims expressed in this article are solely those of the authors and do not necessarily represent those of their affiliated organizations, or those of the publisher, the editors and the reviewers. Any product that may be evaluated in this article, or claim that may be made by its manufacturer, is not guaranteed or endorsed by the publisher.

## References

[B1] Austin P. C. (2011). An introduction to propensity score methods for reducing the effects of confounding in observational studies. *Multivariate Behav. Res.* 46 399–424. 10.1080/00273171.2011.568786 21818162 PMC3144483

[B2] Brookhart M. A. Schneeweiss S. Rothman K. J. Glynn R. J. Avorn J. Sturmer T. (2006). Variable selection for propensity score models. *Am. J. Epidemiol.* 163 1149–1156. 10.1093/aje/kwj149 16624967 PMC1513192

[B3] Calcutt N. A. (2020). Diabetic neuropathy and neuropathic pain: a (con)fusion of pathogenic mechanisms? *Pain* 161(Suppl. 1) S65–S86. 10.1097/j.pain.0000000000001922 32999525 PMC7521457

[B4] Chen L. Islam R. M. Wang J. Hird T. R. Pavkov M. E. Gregg E. W. (2020). A systematic review of trends in all-cause mortality among people with diabetes. *Diabetologia* 63 1718–1735. 10.1007/s00125-020-05199-0 32632526 PMC11000245

[B5] Cheung K. S. Chan E. W. Chen L. Seto W. K. Wong I. C. K. Leung W. K. (2019). Diabetes increases risk of gastric cancer after eradication: a territory-wide study with propensity score analysis. *Diabetes care* 42 1769–1775. 10.2337/dc19-0437 31296646

[B6] Emerging Risk Factors Collaboration, Di Angelantonio E. Kaptoge S. Wormser D. Willeit P. Butterworth A. S. (2015). Association of cardiometabolic multimorbidity with mortality. *JAMA* 314 52–60. 10.1001/jama.2015.7008 26151266 PMC4664176

[B7] Feldman E. L. Callaghan B. C. Pop-Busui R. Zochodne D. W. Wright D. E. Bennett D. L. (2019). Diabetic neuropathy. *Nat. Rev. Dis. Primers* 5:41. 10.1038/s41572-019-0092-1 31197153

[B8] Feldman E. L. Nave K. A. Jensen T. S. Bennett D. L. H. (2017). New horizons in diabetic neuropathy: mechanisms, bioenergetics, and pain. *Neuron* 93 1296–1313. 10.1016/j.neuron.2017.02.005 28334605 PMC5400015

[B9] GBD 2019 Diseases and Injuries Collaborators (2020). Global burden of 369 diseases and injuries in 204 countries and territories, 1990-2019: a systematic analysis for the global burden of disease study 2019. *Lancet* 396 1204–1222. 10.1016/s0140-6736(20)30925-9 33069326 PMC7567026

[B10] Grool A. Aglipay M. Momoli F. Meehan W. Freedman S. Yeates K. (2016). Association between early participation in physical activity following acute concussion and persistent postconcussive symptoms in children and adolescents. *JAMA* 316 2504–2514. 10.1001/jama.2016.17396 27997652

[B11] Haukoos J. S. Lewis R. J. (2015). The propensity score. *JAMA* 314 1637–1638. 10.1001/jama.2015.13480 26501539 PMC4866501

[B12] Hu C. Jia W. (2018). Diabetes in China: epidemiology and genetic risk factors and their clinical utility in personalized medication. *Diabetes* 67 3–11. 10.2337/dbi17-0013 29263166

[B13] Kurth T. Walker A. M. Glynn R. J. Chan K. A. Gaziano J. M. Berger K. (2006). Results of multivariable logistic regression, propensity matching, propensity adjustment, and propensity-based weighting under conditions of nonuniform effect. *Am. J. Epidemiol.* 163 262–270. 10.1093/aje/kwj047 16371515

[B14] Li F. Thomas L. E. Li F. (2019). Addressing extreme propensity scores *via* the overlap weights. *Am. J. Epidemiol.* 188 250–257. 10.1093/aje/kwy201 30189042

[B15] Li Y. Teng D. Shi X. Qin G. Qin Y. Quan H. (2020). Prevalence of diabetes recorded in mainland China using 2018 diagnostic criteria from the american diabetes association: national cross sectional study. *BMJ* 369:m997. 10.1136/bmj.m997 32345662 PMC7186854

[B16] Liebeskind D. S. Juttler E. Shapovalov Y. Yegin A. Landen J. Jauch E. C. (2019). Cerebral edema associated with large hemispheric infarction. *Stroke* 50 2619–2625. 10.1161/STROKEAHA.118.024766 31426728

[B17] Mashour G. A. Shanks A. M. Kheterpal S. (2011). Perioperative stroke and associated mortality after noncardiac, nonneurologic surgery. *Anesthesiology* 114 1289–1296. 10.1097/ALN.0b013e318216e7f4 21478735

[B18] Meagher P. Adam M. Civitarese R. Bugyei-Twum A. Connelly K. A. (2018). Heart failure with preserved ejection fraction in diabetes: mechanisms and management. *Can. J. Cardiol.* 34 632–643. 10.1016/j.cjca.2018.02.026 29731023

[B19] POISE Study Group, Devereaux P. J. Yang H. Yusuf S. Guyatt G. Leslie K. (2008). Effects of extended-release metoprolol succinate in patients undergoingwho underwent non-cardiac surgery (POISE trial): a randomised controlled trial. *Lancet* 371 1839–1847. 10.1016/s0140-6736(08)60601-7 18479744

[B20] Pop-Busui R. Boulton A. J. Feldman E. L. Bril V. Freeman R. Malik R. A. (2017). Diabetic neuropathy: a position statement by the american diabetes association. *Diabetes Care* 40 136–154. 10.2337/dc16-2042 27999003 PMC6977405

[B21] Rawshani A. Rawshani A. Franzen S. Sattar N. Eliasson B. Svensson A. M. (2018). Risk factors, mortality, and cardiovascular outcomes in patients with type 2 diabetes. *N. Engl. J. Med.* 379 633–644. 10.1056/NEJMoa1800256 30110583

[B22] Rich J. T. Neely J. G. Paniello R. C. Voelker C. C. Nussenbaum B. Wang E. W. (2010). A practical guide to understanding Kaplan-Meier curves. *Otolaryngol. Head Neck Surg.* 143 331–336. 10.1016/j.otohns.2010.05.007 20723767 PMC3932959

[B23] Rosenbaum P. R. Rubin D. B. (2012). Constructing a control group using multivariate matched sampling methods that incorporate the propensity score. *Am. Stat.* 39 33–38. 10.1080/00031305.1985.10479383

[B24] Sacco R. L. Kasner S. E. Broderick J. P. Caplan L. R. Connors J. J. Culebras A. (2013). An updated definition of stroke for the 21st century: a statement for healthcare professionals from the american heart association/american stroke association. *Stroke* 44 2064–2089. 10.1161/STR.0b013e318296aeca 23652265 PMC11078537

[B25] Saltman A. P. Silver F. L. Fang J. Stamplecoski M. Kapral M. K. (2015). Care and outcomes of patients with in-hospital stroke. *JAMA Neurol.* 72 749–755. 10.1001/jamaneurol.2015.0284 25938195

[B26] Selim M. (2007). Perioperative stroke. *N. Engl. J. Med.* 356 706–713. 10.1056/NEJMra062668 17301301

[B27] Sheth K. Elm J. Molyneaux B. Hinson H. Beslow L. Sze G. (2016). Safety and efficacy of intravenous glyburide on brain swelling after large hemispheric infarction (GAMES-RP): a randomised, double-blind, placebo-controlled phase 2 trial. *Lancet Neurol.* 15 1160–1169. 10.1016/s1474-4422(16)30196-x 27567243

[B28] Sheth K. N. Petersen N. H. Cheung K. Elm J. J. Hinson H. E. Molyneaux B. J. (2018). Long-term outcomes in patients aged </=70 years with intravenous glyburide from the phase II GAMES-RP study of large hemispheric infarction: an exploratory analysis. *Stroke* 49 1457–1463. 10.1161/STROKEAHA.117.020365 29789393 PMC6192530

[B29] Sloan G. Selvarajah D. Tesfaye S. (2021). Pathogenesis, diagnosis and clinical management of diabetic sensorimotor peripheral neuropathy. *Nat. Rev. Endocrinol.* 17 400–420. 10.1038/s41574-021-00496-z 34050323

[B30] Thiebaut A. M. Gauberti M. Ali C. Martinez De Lizarrondo S. Vivien D. Yepes M. (2018). The role of plasminogen activators in stroke treatment: fibrinolysis and beyond. *Lancet Neurol.* 17 1121–1132. 10.1016/S1474-4422(18)30323-5 30507392

[B31] Vasivej T. Sathirapanya P. Kongkamol C. (2016). Incidence and risk factors of perioperative stroke in noncardiac, and nonaortic and its major branches surgery. *J. Stroke Cerebrovasc. Dis.* 25 1172–1176. 10.1016/j.jstrokecerebrovasdis.2016.01.051 26922129

[B32] Vlisides P. Mashour G. A. (2016). Perioperative stroke. *Can. J. Anaesth.* 63 193–204. 10.1007/s12630-015-0494-9 26391795 PMC4720532

[B33] Vlisides P. E. Moore L. E. (2021). Stroke in surgical patients. *Anesthesiology* 134 480–492. 10.1097/ALN.0000000000003664 33411913

[B34] Vorasayan P. Bevers M. B. Beslow L. A. Sze G. Molyneaux B. J. Hinson H. E. (2019). Intravenous glibenclamide reduces lesional water uptake in large hemispheric infarction. *Stroke* 50 3021–3027. 10.1161/STROKEAHA.119.026036 31537189 PMC6817419

[B35] Wang R. Serruys P. W. Gao C. Hara H. Takahashi K. Ono M. (2021). Ten-year all-cause death after percutaneous or surgical revascularization in diabetic patients with complex coronary artery disease. *Eur. Heart J.* 18:ehab441. 10.1093/eurheartj/ehab441 34405232 PMC8720143

[B36] Wijdicks E. F. M. Sheth K. N. Carter B. S. Greer D. M. Kasner S. E. Kimberly W. T. (2014). Recommendations for the management of cerebral and cerebellar infarction with swelling: a statement for healthcare professionals from the American heart association/American stroke association. *Stroke* 45 1222–1238. 10.1161/01.str.0000441965.15164.d6 24481970

[B37] Williamson E. J. Forbes A. (2014). Introduction to propensity scores. *Respirology* 19 625–635. 10.1111/resp.12312 24889820

[B38] Wong G. Y. Warner D. O. Schroeder D. R. Offord K. P. Warner M. A. Maxson P. M. (2000). Risk of surgery and anesthesia for ischemic stroke. *Anesthesiology* 92 425–432. 10.1097/00000542-200002000-00024 10691229

[B39] Woo S. H. Marhefka G. D. Cowan S. W. Ackermann L. (2021). Development and validation of a prediction model for stroke, cardiac, and mortality risk after non-cardiac surgery. *J. Am. Heart Assoc.* 10:e018013. 10.1161/JAHA.120.018013 33522252 PMC7955339

[B40] Zhang H. Yang L. Zhu X. Zhu M. Sun Z. Cata J. P. (2020). Association between intraoperative intravenous lidocaine infusion and survival in patients undergoingwho underwent pancreatectomy for pancreatic cancer: a retrospective study. *Br. J. Anaesth.* 125 141–148. 10.1016/j.bja.2020.03.034 32475684

