# Corrigendum: Type 2 diabetes increases risk of unfavorable survival outcome for postoperative ischemic stroke in patients who underwent non-cardiac surgery: A retrospective cohort study

**DOI:** 10.3389/fnagi.2022.1120252

**Published:** 2023-02-16

**Authors:** Faqiang Zhang, Yulong Ma, Yao Yu, Miao Sun, Hao Li, Jingsheng Lou, Jiangbei Cao, Yanhong Liu, Mu Niu, Long Wang, Weidong Mi

**Affiliations:** ^1^School of Medicine, Nankai University, Tianjin, China; ^2^Anesthesia and Operation Center, The First Medical Center, Chinese PLA General Hospital, Beijing, China; ^3^Department of Neurology, The Affiliated Hospital of Xuzhou Medical University, Xuzhou Medical University, Xuzhou, China; ^4^Department of Pain Medicine, The First Medical Center, Chinese PLA General Hospital, Beijing, China

**Keywords:** type 2 diabetes mellitus (type 2 DM), overall survival, perioperative stroke, postoperative complications, large hemispheric infarction (LHI)

In the original article, there was an error in the section Results, Baseline Characteristics of Patients, Paragraph 1. The number of the patients without follow-up data has an input error. The corresponding N number for “patients without follow-up data” was given as 72, but should be 76. The corrected Paragraph 1 appears below.

From January 1, 2008, to August 31, 2019, at Chinese PLA General Hospital, a total of 2,21,541 patients who underwent non-cardiac surgery were included, of whom 484 (0.22%) patients were diagnosed to have an ischemic stroke within 30 days after surgery. After excluding 76 patients without follow-up data, 408 of 484 (84.3%) eligible patients with postoperative ischemic stroke remained in the cohort, of whom 113 (27.7%) had DM (Figure 1). During a median follow-up of 46.2 months (IQR: 21.1, 84.2), the overall all-cause mortality was 49.0% (200/408).

In the original article, there was an error in Figure 1. The number of the patients without follow-up data has an input error. The corresponding N number for “patients without follow-up data” was given as 72, but should be 76. The corrected Figure 1 appears below.

**Figure 1:**
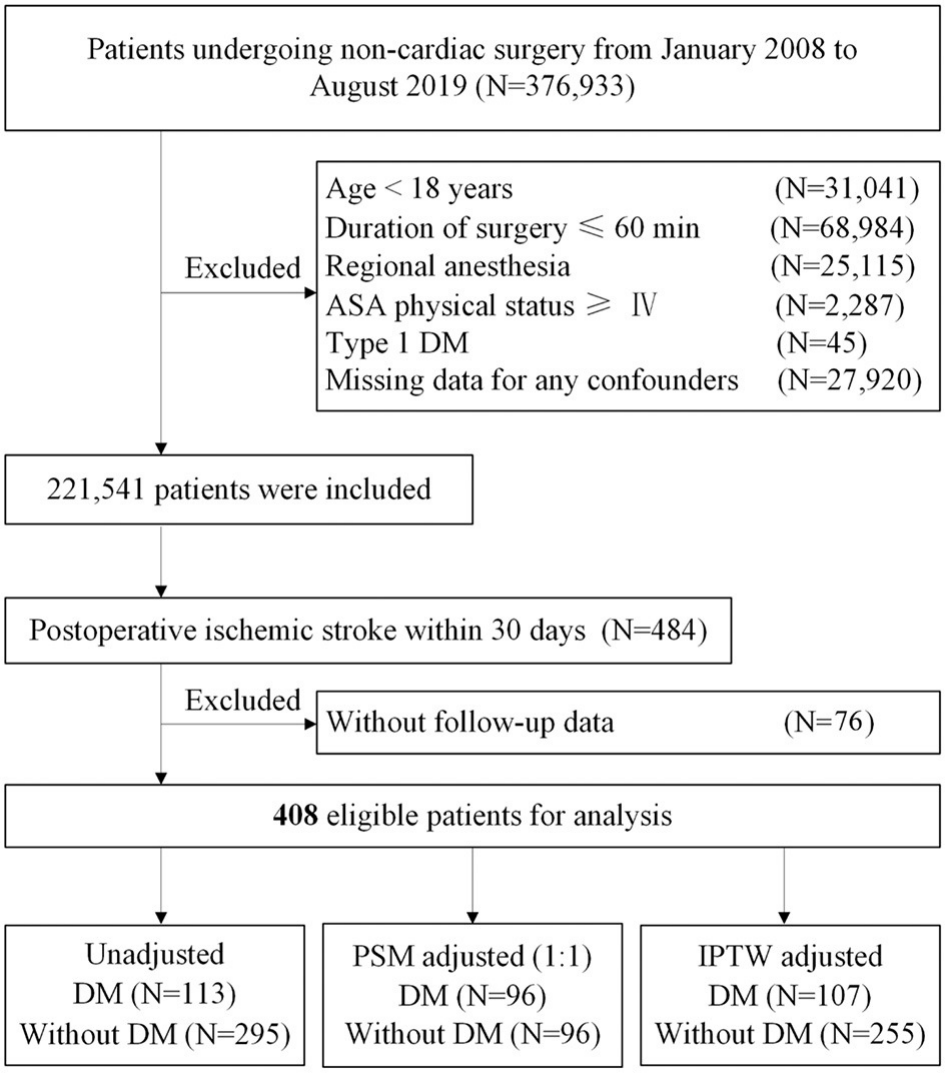
Study flow diagram. ASA, American Society of Anesthesiologists; DM, diabetes mellitus; PSM, propensity score matching; IPTW, inverse probability treatment weighting.

In the original article, there was an error in the section Research Design and Methods, “Exposure of Interest and Covariates,” paragraph 3. The definition of postoperative ischemic stroke was incomplete. The corrected paragraph appears below.

Postoperative ischemic stroke is defined as a brain infarction of ischemic etiology with motor, sensory, or cognitive dysfunction (e.g., hemiplegia, hemiparesis, aphasia, sensory deficit, and impaired memory) 30 days after surgery (Mashour et al., 2011; Sacco et al., 2013; Vlisides and Moore, 2021). Diagnoses of stroke are confirmed by a combination of neuroimaging and clinical evidence of cerebrovascular ischemia during hospital stay. Preoperative MAP was determined on the first blood pressure in the operation room. Stroke laterality, stroke location, and LHI were suggested by CT or MRI. LHI was defined as follow: (1) suffered from >1/3 infarction of the middle cerebral artery (MCA) territory within 6 h after the onset of stroke (or >1/2 MCA territory from 6 h to 7 days after onset) by the assessment of CT scan, (2) exceeded 80 ml within 6 h after the onset of stroke (or 145 ml within 14 h after onset) for infarction volume by appraisal of the MRI), and (3) suffered from >3 cm infarction of cerebellar region on the MRI (Sheth et al., 2016; Vorasayan et al., 2019). In the original article, there was a mistake in the section Discussion, Paragraph 4. Discussion, Paragraph 4 is a duplicate of Discussion, Paragraph 3 and should be deleted.

In the original article, there was a mistake in Figure 4 as published. The death cases of follow-up time >18.5 months subgroup have input errors. The corresponding n number for “Patients without DM and Death cases” was given as 232 (70), but should be 232 (69). In addition, the corresponding n number for “Patients with DM and Death cases” was 81 (39) but should be 81 (42). The revised Figure 4 appears below.

**Figure 4:**
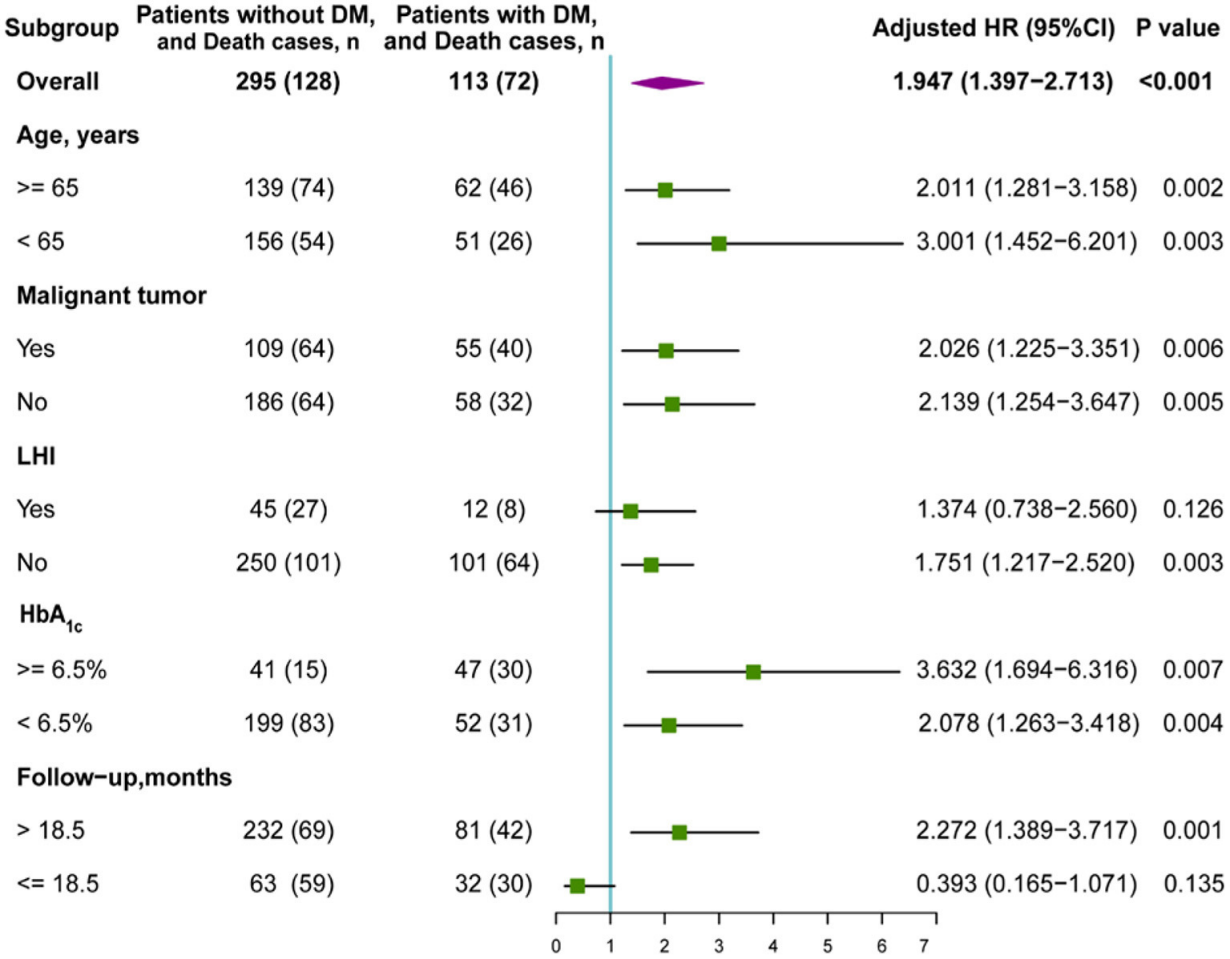
Subgroup analysis of the association between DM and overall survival (OS). HR, hazard ratio; DM, diabetes mellitus; LHI, large hemispheric infarction.

The authors apologize for this error and state that this does not change the scientific conclusions of the article in any way. The original article has been updated.
