# Supplementary material for: Type 2 Diabetes Increases Risk of Unfavorable Survival Outcome for Postoperative Ischemic Stroke in Patients Who Underwent Non-cardiac Surgery: A Retrospective Cohort Study
Source: Front Aging Neurosci. 2022 Jan 11;13:810050. doi: 10.3389/fnagi.2021.810050 (PMC8786912; doi:10.3389/fnagi.2021.810050)
Supplement: Supplementary file 1 [file Data_Sheet_1.doc]

SUPPLEMENTAL MATERIAL

**Supplementary Table 1**. STROBE Statement—Checklist of items that should be included in reports of *cohort studies*

|  | Item  No | Recommendation | Page  No |
| --- | --- | --- | --- |
| **Title and abstract** | 1 | (*a*) Indicate the study’s design with a commonly used term in the title or the abstract | 1,2 |
|  |  | (*b*) Provide in the abstract an informative and balanced summary of what was done and what was found | 2 |
| Introduction | | |  |
| Background/rationale | 2 | Explain the scientific background and rationale for the investigation being reported | 2 |
| Objectives | 3 | State specific objectives, including any prespecified hypotheses | 2 |
| Methods | | |  |
| Study design | 4 | Present key elements of study design early in the paper | 2 |
| Setting | 5 | Describe the setting, locations, and relevant dates, including periods of recruitment, exposure, follow-up, and data collection | 2, 3 |
| Participants | 6 | (*a*) Give the eligibility criteria, and the sources and methods of selection of participants. Describe methods of follow-up | 2, Figure 1 |
|  |  | (*b*) For matched studies, give matching criteria and number of exposed and unexposed | Not applicable |
| Variables | 7 | Clearly define all outcomes, exposures, predictors, potential confounders, and effect modifiers. Give diagnostic criteria, if applicable | 2, 3 Table 1 |
| Data sources/ measurement | 8* | For each variable of interest, give sources of data and details of methods of assessment (measurement). Describe comparability of assessment methods if there is more than one group | 2, 3 Figure 1 |
| Bias | 9 | Describe any efforts to address potential sources of bias | 3 |
| Study size | 10 | Explain how the study size was arrived at | 3, Figure 1 |
| Quantitative variables | 11 | Explain how quantitative variables were handled in the analyses. If applicable, describe which groupings were chosen and why | 3 |
| Statistical methods | 12 | (*a*) Describe all statistical methods, including those used to control for confounding | 3, Table 2 |
|  |  | (*b*) Describe any methods used to examine subgroups and interactions | 3, Figure 4 |
|  |  | (*c*) Explain how missing data were addressed | 2, Figure 1 |
|  |  | (*d*) If applicable, explain how loss to follow-up was addressed | Not applicable |
|  |  | (*e*) Describe any sensitivity analyses | 3, Table 2 |
| Results | | |  |
| Participants | 13* | (a) Report numbers of individuals at each stage of study—eg numbers potentially eligible, examined for eligibility, confirmed eligible, included in the study, completing follow-up, and analysed | 3, Figure 1 |
|  |  | (b) Give reasons for non-participation at each stage | 3, Figure 1 |
|  |  | (c) Consider use of a flow diagram | Figure 1 |
| Descriptive data | 14* | (a) Give characteristics of study participants (eg demographic, clinical, social) and information on exposures and potential confounders | 3, Table 1 |
|  |  | (b) Indicate number of participants with missing data for each variable of interest | Not applicable |
|  |  | (c) Summarise follow-up time (eg, average and total amount) | 5, Table 1 |
| Outcome data | 15* | Report numbers of outcome events or summary measures over time | 5, Table 1, Supplementary Table S3 |
| Main results | 16 | (*a*) Give unadjusted estimates and, if applicable, confounder-adjusted estimates and their precision (eg, 95% confidence interval). Make clear which confounders were adjusted for and why they were included | 5, 6, Table 2,  Figure 2  Figure 3  Supplementary Table S4 |
|  |  | (*b*) Report category boundaries when continuous variables were categorized | Not applicable |
|  |  | (*c*) If relevant, consider translating estimates of relative risk into absolute risk for a meaningful time period | Not applicable |
| Other analyses | 17 | Report other analyses done—eg analyses of subgroups and interactions, and sensitivity analyses | 7,8, Table 2, Figure 4,  Supplementary Table S5, Table S6 |
| Discussion | | |  |
| Key results | 18 | Summarise key results with reference to study objectives | 8 |
| Limitations | 19 | Discuss limitations of the study, taking into account sources of potential bias or imprecision. Discuss both direction and magnitude of any potential bias | 9, 10 |
| Interpretation | 20 | Give a cautious overall interpretation of results considering objectives, limitations, multiplicity of analyses, results from similar studies, and other relevant evidence | 8, 9 |
| Generalisability | 21 | Discuss the generalisability (external validity) of the study results | 9 |
| Other information | | |  |
| Funding | 22 | Give the source of funding and the role of the funders for the present study and, if applicable, for the original study on which the present article is based | 10 |

*Give information separately for exposed and unexposed groups.

**Note:** An Explanation and Elaboration article discusses each checklist item and gives methodological background and published examples of transparent reporting. The STROBE checklist is best used in conjunction with this article (freely available on the Web sites of PLoS Medicine at http://www.plosmedicine.org/, Annals of Internal Medicine at http://www.annals.org/, and Epidemiology at http://www.epidem.com/). Information on the STROBE Initiative is available at http://www.strobe-statement.org.

**Supplementary Table 2**. Comorbidity and outcome definitions based on International Classification of Diseases, Ninth/Tenth Revision (ICD-9/10) codes.

| Comorbidity | Code type | Code | Description |
| --- | --- | --- | --- |
| Diabetes  mellitus with  and without  chronic  complications | ICD-10 | E10.0X | Type 1 DM |
|  | ICD-10 | E10.1X | Type 1 DM with ketoacidosis |
|  | ICD-10 | E10.6X | Type 1 DM with other specified complications |
|  | ICD-10 | E10.8X | Type 1 DM with unspecified complications |
|  | ICD-10 | E10.9X | Type 1 DM without complications |
|  | ICD-10 | E11.0X | Type 2 DM with hyperosmolarity |
|  | ICD-10 | E11.1X | Type 2 DM with hyperosmolarity with coma |
|  | ICD-10 | E11.6X | Type 2 DM with other specified complications |
|  | ICD-10 | E11.8X | Type 2 DM with unspecified complications |
|  | ICD-10 | E11.9X | Type 2 DM without complications |
|  | ICD-10 | E12.0X | DM associated with malnutrition |
|  | ICD-10 | E12.1X | DM associated with malnutrition with coma |
|  | ICD-10 | E12.6X | DM associated with malnutrition with other specified  complications |
|  | ICD-10 | E12.8X | DM associated with malnutrition with unspecified complications |
|  | ICD-10 | E12.9X | DM associated with malnutrition without complications |
|  | ICD-10 | E13.0X | Other specified DM with hyperosmolarity |
|  | ICD-10 | E13.1X | Other specified DM with ketoacidosis |
|  | ICD-10 | E13.6X | Other specified DM with other specified complications |
|  | ICD-10 | E13.8X | Other specified DM with unspecified complications |
|  | ICD-10 | E13.9X | Other specified DM without complications |
|  | ICD-10 | E14.0X | DM, not elsewhere classified, with coma |
|  | ICD-10 | E14.1X | DM, not elsewhere classified, with ketoacidosis |
|  | ICD-9/ICD-10 | 250.8/E14.6X | Unspecified DM with other specified complications |
|  | ICD-9/ICD-10 | 250.9/E14.8X | Unspecified DM with other specified complications |
|  | ICD-10 | E14.9X | DM, not elsewhere classified, without complications |
|  | ICD-9 | 250.1 | DM with ketoacidosis |
|  | ICD-9 | 250.0 | DM without complications |
|  | ICD-9 | 250.2 | DM with hyperosmolarity |
|  | ICD-9 | 250.3 | DM with other coma |
|  | ICD-10 | E10.2X | Type 1 DM with kidney complications |
|  | ICD-10 | E10.3X | Type 1 DM with ophthalmic complications |
|  | ICD-10 | E10.4X | Type 1 DM with neurological complications |
|  | ICD-10 | E10.5X | Type 1 DM with circulatory complications |
|  | ICD-10 | E10.7X | Type 1 DM with multiple complications |
|  | ICD-10 | E11.2X | Type 2 DM with kidney complications |
|  | ICD-10 | E11.3X | Type 2 DM with ophthalmic complications |
|  | ICD-10 | E11.4X | Type 2 DM with neurological complications |
|  | ICD-10 | E11.5X | Type 2 DM with circulatory complications |
|  | ICD-10 | E11.7X | Type 2 DM with multiple complications |
|  | ICD-10 | E12.2X | DM associated with malnutrition with renal complications |
|  | ICD-10 | E12.3X | DM associated with malnutrition with ophthalmic complications |
|  | ICD-10 | E12.4X | DM associated with malnutrition with neurological complications |
|  | ICD-10 | E12.5X | DM associated with malnutrition with peripheral vascular complications |
|  | ICD-10 | E12.7X | DM associated with malnutrition with multiple complications |
|  | ICD-10 | E13.2X | Other specified DM with kidney complications |
|  | ICD-10 | E13.3X | Other specified DM with ophthalmic complications |
|  | ICD-10 | E13.4X | Other specified DM with neurological complications |
|  | ICD-10 | E13.5X | Other specified DM with circulatory complications |
|  | ICD-10 | E13.7X | Other specified DM with multiple complications |
|  | ICD-10 | E14.2X | DM, not elsewhere specified, with renal complications |
|  | ICD-10 | E14.3X | DM, not elsewhere specified, with ophthalmic complications |
|  | ICD-10 | E14.4X | DM, not elsewhere specified, with neurological complications |
|  | ICD-10 | E14.5X | DM, not elsewhere specified, with peripheral vascular  complications |
|  | ICD-10 | E14.7X | DM, not elsewhere specified, with multiple complications |
|  | ICD-9 | 250.4 | Diabetes with renal complications |
|  | ICD-9 | 250.5 | Diabetes with ophthalmic complications |
|  | ICD-9 | 250.6 | Diabetes with neurological complications |
|  | ICD-9 | 250.7 | Diabetes with peripheral circulatory disorders |
| Hypertension | ICD-9/ICD-10 | 401.X/I10.X | Essential hypertension |
| Myocardial  infarction | ICD-10 | I22.X | Recurring myocardial infarction |
|  | ICD-9/ICD-10 | 412.X/I25.2X | Old/healed myocardial infarction |
| Peripheral  vascular  disease | ICD-9/ICD-10 | 440.X/I70.X | Atherosclerosis |
|  | ICD-9/ICD-10 | 441.X/I71.X | Aortic aneurysm and dissection |
|  | ICD-9/ICD-10 | 443.1/I73.1 | Thromboangiitis obliterans (Buerger’s disease) |
|  | ICD-9/ICD-10 | 443.8X/I73.8X | Other specified peripheral vascular diseases |
|  | ICD-9/ICD-10 | 443.9/I73.9 | Peripheral vascular disease, unspecified |
|  | ICD-9/ICD-10 | 447.1/I77.1 | Stricture of artery |
|  | ICD-10 | I79.0X | Aneurysm of aorta in diseases classified elsewhere |
|  | ICD-10 | I79.2X | Peripheral angiopathy |
|  | ICD-9/ICD-10 | 557.1/K55.1X | Chronic vascular disorders of intestine |
|  | ICD-10 | K55.8X | Other vascular disorders of intestine |
|  | ICD-9/ICD-10 | 557.9/K55.9X | Vascular disorders of intestine, unspecified |
|  | ICD-9/ICD-10 | V43.4/Z95.8X | Presence of other cardiac and vascular implants and grafts |
|  | ICD-10 | Z95.9X | Presence of cardiac and vascular implant and graft,unspecified |
|  | ICD-9 | 442.X | Other aneurysm |
|  | ICD-9 | 443.2X | Other arterial dissection |
|  | ICD-9/ICD-10 | 440.2, I70.2 | Peripheral arterial disease |
|  | ICD-9/ICD-10 | 440.3, I70.3,  I70.5, I70.6,  I70.7 | Atherosclerosis of bypass graft of the extremities |
|  | ICD-9/ICD-10 | 440.4, I70.92 | Chronic total occlusion of artery of the extremities |
|  | ICD-9 | 443.9 | Peripheral vascular disease, unspecified |
| Ischemic stroke | ICD-9/ICD-10 | 433.X1/I63.X | Occlusion and stenosis of precerebral arteries with cerebral  infarction |
|  | ICD-9 | 434.X1 | Occlusion of cerebral arteries with cerebral infarction |
|  | ICD-9/ICD-10 | 437.1/I67.81,  I67.89 | Other generalized ischemic cerebrovascular disease |
|  | ICD-9/ICD-10 | 437.9/I67.9 | Unspecified cerebrovascular disease |

**Supplementary Table 3** Causes of death of postoperative ischemic stroke patients.

| Cause of death^*^ | ALL  NO. % | | Patients without DM  NO. % | | Patients with DM  NO. % | |
| --- | --- | --- | --- | --- | --- | --- |
| Cerebrovascular diseases^†^ | 67 | 35.1 | 36 | 29.5 | 31 | 44.9 |
| Cardiovascular diseases^‡^ | 43 | 22.5 | 24 | 19.7 | 19 | 27.5 |
| Cancer | 50 | 26.1 | 37 | 30.3 | 13 | 18.8 |
| Other diseases^§^ | 27 | 14.2 | 22 | 18.0 | 5 | 7.4 |
| Accidents | 4 | 2.1 | 3 | 2.5 | 1 | 1.4 |
| Total | 191 | 100 | 122 | 100 | 69 | 100 |
| Abbreviations: DM, diabetes mellitus.  ^*^Unknown in 9 cases.  ^†^ Cerebrovascular diseases included subarachnoid haemorrhage, cerebral haemorrhage, cerebral thrombosis, cerebral embolism, occlusion of precerebral arteries.  ^‡^ Cardiovascular diseases included acute myocardial infarction, hypertensive heart disease, hypertensive renal disease, arterial embolism and thrombosis, gangrene, aortic aneurysm, pulmonary embolism and infarction, portal vein thrombosis, venous embolism and thrombosis.  ^§^ Other diseases included pneumonia, emphysema, cirrhosis of liver, acute pancreatitis, acute renal failure. | | | | | | |

**Supplementary Table 4** Univariate and multivariate Cox regression analyses for overall survival (OS) in the Model 5.

| Variables | Univariate analysis | | Multivariate analysis | |
| --- | --- | --- | --- | --- |
|  | HR (95% CI) | *P* value | HR (95% CI) | *P* value |
| DM (Yes vs No) | 1.763 (1.319-2.356) | <0.001 | 1.947 (1.397-2.713) | <0.001 |
| Age | 1.027 (1.013-1.040) | <0.001 | 1.021 (1.007-1.035) | 0.003 |
| Sex (male vs female) | 0.821 (0.620-1.088) | 0.170 |  |  |
| BMI | 0.949 (0.911-0.988) | 0.011 | 0.981 (0.931-1.034) | 0.486 |
| ASA physical status |  |  |  |  |
| Class Ⅰ | reference |  |  |  |
| Class Ⅱ | 1.535 (0.626-3.764) | 0.349 |  |  |
| Class Ⅲ | 1.776 (0.714-4.420) | 0.217 |  |  |
| Hypertension (Yes vs No) | 0.841 (0.636-1.111) | 0.222 |  |  |
| Previous ischemic stroke (Yes vs No) | 1.257 (0.940-1.681) | 0.123 |  |  |
| Myocardial infarction (Yes vs No) | 1.664 (1.068-2.593) | 0.024 | 1.094 (0.613-1.952) | 0.761 |
| Peripheral vascular disease (Yes vs No) | 0.857 (0.602-1.220) | 0.391 |  |  |
| Chronic kidney disease (Yes vs No) | 1.512 (0.671-3.409) | 0.319 |  |  |
| Preoperative β blockers (Yes vs No) | 0.986 (0.627-1.551) | 0.952 |  |  |
| Preoperative aspirin (Yes vs No) | 1.077 (0.802-1.446) | 0.623 |  |  |
| Preoperative Hb | 0.991 (0.984-0.998) | 0.017 | 1.001 (0.991-1.011) | 0.892 |
| Preoperative TBIL | 1.007 (1.002-1.011) | 0.002 | 1.004 (0.998-1.009) | 0.204 |
| Preoperative PT | 1.081 (0.987-1.183) | 0.094 |  |  |
| Preoperative NLR | 1.036 (1.008-1.064) | 0.012 | 1.090 (1.026-1.160) | 0.006 |
| Preoperative PLR | 1.002 (1.001-1.003) | 0.002 | 1.003 (1.001-1.005) | 0.013 |
| Emergency surgery (Yes vs No) | 1.254 (0.824-1.908) | 0.290 |  |  |
| Surgical service |  |  |  |  |
| Spine | reference |  | reference |  |
| Intra-abdominal surgery | 2.320 (1.368-3.936) | 0.002 | 1.022 (0.505-2.069) | 0.951 |
| Joint arthroplary | 0.877 (0.429-1.794) | 0.719 | 0.808 (0.378-1.724) | 0.581 |
| Oral and maxillofacial | 2.175 (1.062-4.454) | 0.034 | 1.583 (0.672-3.726) | 0.293 |
| Urologic | 1.407 (0.744-2.660) | 0.294 | 0.764 (0.345-1.689) | 0.506 |
| Neurosurgery | 1.158 (0.700-1.917) | 0.568 | 1.175 (0.604-2.286) | 0.635 |
| Thoracic or vascular | 2.126 (0.994-4.548) | 0.052 | 1.007 (0.385-2.634) | 0.989 |
| Other (ENT, etc) | 0.999 (0.541-1.843) | 0.998 | 0.881 (0.426-1.822) | 0.732 |
| Malignant tumor (Yes vs No) | 2.112 (1.597-2.793) | <0.001 | 2.044 (1.528-2.735) | <0.001 |
| Duration of surgery | 1.001 (1.000-1.002) | 0.102 |  |  |
| Estimated blood loss | 1.003 (1.001-1.005) | 0.004 | 0.998 (0.994-1.001) | 0.296 |
| Preoperative MAP | 0.997 (0.986-1.008) | 0.603 |  |  |
| Blood products depot (Yes vs No) | 2.119 (1.556-2.885) | <0.001 | 1.929 (1.405-2.648) | <0.001 |
| Colloids infusion | 2.800 (0.081-4.809) | 0.569 |  |  |
| Crystalloids infusion | 1.352 (0.889-3.513) | 0.063 |  |  |
| NSAIDs (Yes vs No) | 1.367 (0.905-2.064) | 0.138 |  |  |
| Morphine equivalents | 1.000 (0.998-1.003) | 0.879 |  |  |
| ICU admission after surgery (Yes vs No) | 1.518 (1.149-2.006) | 0.003 | 1.385 (0.941-2.038) | 0.099 |
| Stroke severity (NIHSS) | 1.126 (1.019-1.327) | 0.029 | 1.101 (0.875-1.985) | 0.098 |
| Stroke laterality |  |  |  |  |
| Left | reference |  | reference |  |
| Right | 1.103 (0.788-1.544) | 0.567 | 1.066 (0.737-1.541) | 0.734 |
| Bilateral | 1.577 (1.118-2.223) | 0.009 | 1.413 (0.931-2.144) | 0.105 |
| Stroke location |  |  |  |  |
| Cortical | reference |  | reference |  |
| Subcortical | 0.597 (0.415-0.857) | 0.005 | 0.651 (0.410-1.035) | 0.070 |
| Cerebellar | 0.764 (0.237-2.461) | 0.651 | 0.448 (0.121-1.664) | 0.230 |
| Brainstem | 1.121 (0.272-4.626) | 0.875 | 0.919 (0.189-4.483) | 0.918 |
| Multiple | 0.782 (0.531-1.151) | 0.213 | 0.682 (0.420-1.107) | 0.122 |
| LHI (Yes vs No) | 1.832 (1.291-2.600) | <0.001 | 2.523 (1.736-3.667) | <0.001 |
| Thrombolysis (Yes vs No) | 0.998 (0.856-1.315) | 0.215 |  |  |
| Abbreviations: HR, hazard ratio; DM, diabetes mellitus; BMI, body mass index; ASA, American Society of Anesthesiologists; Hb, hemoglobin; ALB, albumin; TBIL, total bilirubin; PT, prothrombin time; NLR, neutrophil-lymphocyte ratio; PLR, platelet to lymphocyte ratio; ENT, ear, nose and throat; MAP, mean arterial pressure; NSAIDs, nonsteroid anti-inflammatory drugs; ICU, intensive care unit; NIHSS, national institutes of health stroke scale; LHI, large hemispheric infarction; CI, confidence interval. | | | | |

**Supplementary Table 5** Univariate and multivariate Cox regression analyses for overall survival (OS) in the PS matching.

| Variables | Univariate analysis | | Multivariate analysis | |
| --- | --- | --- | --- | --- |
|  | HR (95% CI) | *P* value | HR (95% CI) | *P* value |
| DM (Yes vs No) | 1.771 (1.178-2.661) | <0.001 | 2.190 (1.354-3.540) | 0.001 |
| Age | 1.017 (1.002-1.025) | 0.002 | 1.020 (0.991-1.050) | 0.178 |
| Sex (male vs female) | 0.648 (0.430-0.977) | 0.038 | 0.823 (0.459-1.478) | 0.515 |
| BMI | 0.922 (0.873-0.974) | 0.626 |  |  |
| ASA physical status |  |  |  |  |
| Class Ⅰ | reference |  |  |  |
| Class Ⅱ | 3.155 (0.436-5.851) | 0.255 |  |  |
| Class Ⅲ | 2.844 (0.389-5.81) | 0.303 |  |  |
| Hypertension (Yes vs No) | 0.572 (0.384-0.853) | 0.006 | 0.606 (0.353-1.040) | 0.069 |
| Previous ischemic stroke (Yes vs No) | 1.168 (0.783-1.742) | 0.448 |  |  |
| Myocardial infarction (Yes vs No) | 1.418 (0.818-2.460) | 0.214 |  |  |
| Peripheral vascular disease (Yes vs No) | 0.751 (0.459-1.230) | 0.255 |  |  |
| Chronic kidney disease (Yes vs No) | 0.586 (0.081-4.210) | 0.595 |  |  |
| Preoperative β blockers (Yes vs No) | 0.902 (0.481-1.693) | 0.749 |  |  |
| Preoperative aspirin (Yes vs No) | 0.987 (0.654-1.491) | 0.952 |  |  |
| Preoperative Hb | 0.993 (0.982-1.005) | 0.257 |  |  |
| Preoperative TBIL | 1.004 (0.996-1.012) | 0.310 |  |  |
| Preoperative PT | 1.082 (0.958-1.223) | 0.205 |  |  |
| Preoperative NLR | 1.020 (1.005-1.082) | 0.026 | 1.110 (1.046-1.188) | 0.001 |
| Preoperative PLR | 1.004 (1.001-1.007) | 0.039 | 1.005 (0.998-1.007) | 0.073 |
| Emergency surgery (Yes vs No) | 0.743 (0.344-1.607) | 0.451 |  |  |
| Surgical service |  |  |  |  |
| Spine | reference |  | reference |  |
| Intra-abdominal surgery | 2.276 (1.108-4.678) | 0.025 | 0.955 (0.306-2.984) | 0.937 |
| Joint arthroplary | 1.444 (0.493-4.234) | 0.503 | 1.472 (0.429-5.055) | 0.539 |
| Oral and maxillofacial | 4.363 (1.573-6.099) | 0.005 | 0.979 (0.230-4.172) | 0.977 |
| Urologic | 1.832 (0.791-4.246) | 0.158 | 0.764 (0.207-2.688) | 0.654 |
| Neurosurgery | 1.135 (0.531-2.426) | 0.744 | 1.305 (0.442-3.858) | 0.630 |
| Thoracic or vascular | 1.717 (0.538-5.478) | 0.361 | 0.609 (0.385-3.040) | 0.545 |
| Other (ENT, etc) | 2.020 (0.857-4.76) | 0.108 | 1.770 (0.454-6.899) | 0.411 |
| Malignant tumor (Yes vs No) | 2.267 (1.505-3.415) | <0.001 | 2.416 (1.995-5.866) | <0.001 |
| Duration of surgery | 1.001 (1.000-1.002) | 0.102 |  |  |
| Estimated blood loss | 1.005 (1.002-1.009) | 0.164 |  |  |
| Preoperative MAP | 0.996 (0.978-1.015) | 0.670 |  |  |
| Blood products depot (Yes vs No) | 2.376 (1.492-3.785) | <0.001 | 1.682 (0.826-3.425) | 0.151 |
| Colloids infusion | 2.652 (1.081-5.809) | 0.886 |  |  |
| Crystalloids infusion | 1.152 (0.689-4.513) | 0.063 |  |  |
| NSAIDs (Yes vs No) | 0.982 (0.605-1.594) | 0.942 |  |  |
| Morphine equivalents | 0.999 (0.995-1.002) | 0.478 |  |  |
| ICU admission after surgery (Yes vs No) | 1.286 (0.863-1.915) | 0.216 |  |  |
| Stroke severity (NIHSS) | 1.229 (1.056-1.712) | 0.016 | 1.323 (0.812-2.115) | 0.065 |
| Stroke laterality |  |  |  |  |
| Left | reference |  |  |  |
| Right | 0.872 (0.542-1.403) | 0.572 |  |  |
| Bilateral | 1.387 (0.851-2.261) | 0.190 |  |  |
| Stroke location |  |  |  |  |
| Cortical | reference |  |  |  |
| Subcortical | 0.733 (0.392-1.371) | 0.331 |  |  |
| Cerebellar | 0.415 (0.054-3.199) | 0.399 |  |  |
| Brainstem | 1.426 (0.578-4.242) | 0.996 |  |  |
| Multiple | 0.918 (0.468-1.800) | 0.802 |  |  |
| LHI (Yes vs No) | 1.478 (1.188-2.773) | <0.001 | 3.871 (1.710-8.763) | 0.001 |
| Thrombolysis (Yes vs No) ^a^ | 0.901 (0.854-1.411) |  |  |  |
| Abbreviations: HR, hazard ratio; DM, diabetes mellitus; BMI, body mass index; ASA, American Society of Anesthesiologists; Hb, hemoglobin; ALB, albumin; TBIL, total bilirubin; PT, prothrombin time; NLR, neutrophil-lymphocyte ratio; PLR, platelet to lymphocyte ratio; ENT, ear, nose and throat; MAP, mean arterial pressure; NSAIDs, nonsteroid anti-inflammatory drugs; ICU, intensive care unit; NIHSS, national institutes of health stroke scale; LHI, large hemispheric infarction; CI, confidence interval. | | | | |

**Supplementary Table 6** Univariate and multivariate Cox regression analyses for overall survival (OS) in the IPTW.

| Variables | Univariate analysis | | Multivariate analysis | |
| --- | --- | --- | --- | --- |
|  | HR (95% CI) | *P* value | HR (95% CI) | *P* value |
| DM (Yes vs No) | 1.907 (1.385-2.626) | <0.001 | 2.550 (1.769-3.679) | <0.001 |
| Age | 1.020 (1.002-1.038) | 0.007 | 1.014 (0.990-1.039) | 0.241 |
| Sex (male vs female) | 0.910 (0.658-1.258) | 0.315 |  |  |
| BMI | 0.939 (0.898-1.082) | 0.502 |  |  |
| ASA physical status |  |  |  |  |
| Class Ⅰ | reference |  |  |  |
| Class Ⅱ | 2.726 (0.652-5.386) | 0.375 |  |  |
| Class Ⅲ | 2.883 (0.683-5.162) | 0.442 |  |  |
| Hypertension (Yes vs No) | 0.702 (0.508-1.169) | 0.082 |  |  |
| Previous ischemic stroke (Yes vs No) | 1.023 (0.734-1.426) | 0.133 |  |  |
| Myocardial infarction (Yes vs No) | 1.300 (0.846-1.997) | 0.197 |  |  |
| Peripheral vascular disease (Yes vs No) | 0.745 (0.507-1.096) | 0.594 |  |  |
| Chronic kidney disease (Yes vs No) | 1.423 (0.444-4.559) | 0.319 |  |  |
| Preoperative β blockers (Yes vs No) | 1.054 (0.633-1.755) | 0.204 |  |  |
| Preoperative aspirin (Yes vs No) | 0.994 (0.715-1.381) | 0.478 |  |  |
| Preoperative Hb | 0.987 (0.979-0.996) | <0.001 | 1.002 (0.987-1.016) | 0.805 |
| Preoperative TBIL | 1.006 (0.994-1.008) | 0.005 | 1.004 (0.998-1.008) | 0.141 |
| Preoperative PT | 1.053 (0.922-1.202) | 0.759 |  |  |
| Preoperative NLR | 1.012 (1.007-1.059) | 0.012 | 1.171 (1.018-1.199) | 0.009 |
| Preoperative PLR | 1.001 (0.999-1.004) | 0.215 |  |  |
| Emergency surgery (Yes vs No) | 0.714 (0.39-1.307) | 0.412 |  |  |
| Surgical service |  |  |  |  |
| Spine | reference |  | reference |  |
| Intra-abdominal surgery | 1.910 (1.071-3.408) | 0.019 | 1.056 (0.448-2.492) | 0.900 |
| Joint arthroplary | 1.027 (0.464-2.272) | 0.066 | 0.818 (0.387-1.728) | 0.599 |
| Oral and maxillofacial | 2.869 (1.469-5.603) | 0.038 | 1.368 (0.520-3.596) | 0.525 |
| Urologic | 1.606 (0.864-2.989) | 0.497 | 0.769 (0.312-1.899) | 0.569 |
| Neurosurgery | 1.122 (0.630-2.001) | 0.392 | 1.048 (0.479-2.293) | 0.907 |
| Thoracic or vascular | 1.520 (0.642-3.600) | 0.952 | 0.991 (0.234-4.201) | 0.990 |
| Other (ENT,etc) | 1.643 (0.890-3.036) | 0.587 | 1.544 (0.482-4.951) | 0.465 |
| Malignant tumor (Yes vs No) | 1.948 (1.404-2.704) | <0.001 | 2.044 (1.528-2.735) | <0.001 |
| Duration of surgery | 1.001 (0.999-1.002) | 0.179 |  |  |
| Estimated blood loss | 1.003 (0.992-1.012) | 0.138 |  |  |
| Preoperative MAP | 0.998 (0.982-1.014) | 0.458 |  |  |
| Blood products depot (Yes vs No) | 1.837 (1.265-2.667) | <0.001 | 1.929 (1.405-2.648) | <0.001 |
| Colloids infusion | 1.958 (0.034-3.724) | 0.325 |  |  |
| Crystalloids infusion | 2.212 (0.168-4.278) | 0.603 |  |  |
| NSAIDs (Yes vs No) | 0.977 (0.650-1.468) | 0.215 |  |  |
| Morphine equivalents | 1.001 (0.998-1.004) | 0.784 |  |  |
| ICU admission after surgery (Yes vs No) | 1.363 (0.983-1.890) | 0.186 |  |  |
| Stroke severity (NIHSS) | 1.220 (1.186-1.255) | 0.033 | 1.223 (0.775-1.885) | 0.081 |
| Stroke laterality |  |  |  |  |
| Left | reference |  |  |  |
| Right | 1.051 (0.712-1.551) | 0.249 |  |  |
| Bilateral | 1.394 (0.941-2.065) | 0.659 |  |  |
| Stroke location |  |  |  |  |
| Cortical | reference |  | reference |  |
| Subcortical | 0.499 (0.331-0.752) | <0.001 | 0.651 (0.410-1.035) | 0.070 |
| Cerebellar | 0.302 (0.068-1.343) | 0.359 | 0.448 (0.121-1.664) | 0.230 |
| Brainstem | 0.839 (0.112-6.291) | 0.655 | 0.919 (0.189-4.483) | 0.918 |
| Multiple | 0.782 (0.531-1.151) | 0.159 | 0.682 (0.420-1.107) | 0.122 |
| LHI (Yes vs No) | 1.949 (1.314-2.891) | <0.001 | 2.466 (1.459-4.170) | <0.001 |
| Thrombolysis (Yes vs No) | 0.892 (0.816-1.205) | 0.117 |  |  |
| Abbreviations: HR, hazard ratio; DM, diabetes mellitus; BMI, body mass index; ASA, American Society of Anesthesiologists; Hb, hemoglobin; ALB, albumin; TBIL, total bilirubin; PT, prothrombin time; NLR, neutrophil-lymphocyte ratio; PLR, platelet to lymphocyte ratio; ENT, ear, nose and throat; MAP, mean arterial pressure; NSAIDs, nonsteroid anti-inflammatory drugs; ICU, intensive care unit; NIHSS, national institutes of health stroke scale; LHI, large hemispheric infarction; CI, confidence interval. | | | | |
